# Correction: Utilizing FBR to produce olefins from CO reduction using Fe–Mn nanoparticles on reduced graphene oxide catalysts and comparing the performance with SBR

**DOI:** 10.1039/c9ra90024a

**Published:** 2019-04-08

**Authors:** A. L.-Hassan Nasser, Hamada EL-Naggar, Haitham El-Bery, Islam Basha, Ahmed Abdelmoneim

**Affiliations:** Materials Science and Engineering Department, Egypt-Japan University of Science and Technology New Borg El-Arab Alexandria 21934 Egypt codecsubzero@gmail.com ahmed.abdelmoneim@ejust.edu.eg; Chemical Engineering Department, Faculty of Engineering, Alexandria University Alexandria 11432 Egypt; Advanced Functional Materials Laboratory, Chemistry Department, Faculty of Science, Assiut University Assiut 71516 Egypt; Chemistry Department, Faculty of Science, Alexandria University Alexandria 11432 Egypt

## Abstract

Correction for ‘Utilizing FBR to produce olefins from CO reduction using Fe–Mn nanoparticles on reduced graphene oxide catalysts and comparing the performance with SBR’ by AL-Hassan Nasser *et al.*, *RSC Adv.*, 2018, **8**, 42415–42423.

The authors regret that the original author list was incorrect. The correct author list is as shown above.

The Royal Society of Chemistry apologises for these errors and any consequent inconvenience to authors and readers.

## Supplementary Material

